# Supplementary material for: HIVprotI: an integrated web based platform for prediction and design of HIV proteins inhibitors
Source: J Cheminform. 2018 Mar 9;10:12. doi: 10.1186/s13321-018-0266-y (PMC5845081; doi:10.1186/s13321-018-0266-y)
Supplement: Supplementary file 2 — Additional file 2. Source code of HIVProtI web server. [file 13321_2018_266_MOESM2_ESM.zip › HIVprotI_Source-code/hivprotI/jsme/JSME_template.html]

JME template creation


# This page shows how to create custom templates for JSME

To create a template, one has to:

1. draw a chemical structure
2. mark the connection atom using the blue circle
3. save the template as JME string (button "get JME string")

The atom that should act as a connection point (root) of the template should by marked by :1 (for example the oxygen serving as root should have atomic symbol O:1).

You have to enable JavaScript in your browser to use JSME!

get JME string

  


---

To use a predefined template:

1. draw a chemical structure
2. input a name
3. Click on "set template"
4. Click on one atom of the chemical structure to attach the template, or alternatively use the NEW button and click elsewhere in the drawing area

set template   
Template name:   

See this page for real example.
